# Urothermal Synthesis of Luminescent Alkaline Earth MOFs

**DOI:** 10.1002/chem.70996

**Published:** 2026-04-18

**Authors:** Michaël Teixeira, Stéphane A. Baudron

**Affiliations:** ^1^ Laboratoire De Synthèse Et Fonctions des Architectures Moléculaires CNRS‐Université de Strasbourg, CMC UMR Strasbourg France

**Keywords:** alkaline earth metals, luminescence, metal‐organic frameworks, urothermal synthesis

## Abstract

The urothermal approach in 1,3‐dimethylimidazolidin‐2‐one (e‐murea) was employed for the successful preparation of a series of nine alkaline earth (AE) metal‐organic frameworks (AE‐MOFs) with two different dihydroxy terephthalic acid ligands (2,5‐dobdcH_4_ and 2,3‐dobdcH_4_; dobdcH_4_ = dihydroxyterephthalic acid). The compounds were characterized by single‐crystal and powder X‐ray diffraction as well as elemental analysis and infra‐red spectroscopy to confirm their purity. With the 2,5‐dobdcH_2_^2−^ ligand, two isostructural 3D MOFs were obtained with Mg(II) and Ca(II), whereas 2D architectures featuring coordination of the hydroxyl groups were isolated with Sr(II) and Ba(II). Binding of the hydroxyl groups in a chelate fashion is consistently observed in AE‐MOFs incorporating the 2,3‐dobdcH_2_^2−^ ligand. These AE‐MOFs present strong luminescence in the solid state with quantum yields ranging between 0.45 and 0.84, remarkable for this type of compounds. Interestingly for the AE‐MOFs based on the 2,5‐dobdcH_2_^2−^ ligand, a dependence on the nature of the alkaline earth metal cation was observed, as expressed for the heavier Sr(II) and Ba(II) systems in the presence of two different emission processes in thermal equilibrium as confirmed by investigation of the solid‐state luminescence at 77K. For the AE‐MOFs based on the 2,3‐dobdcH_2_^2−^ ligand, such metal‐ and temperature‐dependence is not observed.

## Graphical abstract

The e‐murea derivative (1,3‐dimethylimidazolidin‐2‐one) was employed as solvent for the urothermal synthesis of a series of nine AE MOFs featuring luminescence in the crystalline state with remarkable quantum yields ranging from 0.45 to 0.84.

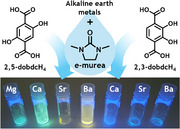

## 1 Introduction

**FIGURE 1:**
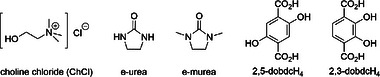
Representation of ChCl and e‐urea, used for DES preparation of MOFs, and of e‐murea envisioned here as an alternative solvent for urothermal synthesis and ligands 2,5‐ and 2,3‐dobdcH_4_ employed in this study.

With a wide range of potential applications in varied fields such as catalysis, sensing, gas storage and separation, among others, Metal‐Organic Frameworks (MOFs) have risen as an important class of materials over the last three decades [1, 2, 3, 4, 5]. These crystalline porous coordination polymers are constructed by assembly of polytopic bridging ligands with metal centers/nodes. The latter play a key role in the structure and properties of the MOFs. While transition metal cations and lanthanides have been extensively explored, MOFs based on alkaline earth (AE) metals have been comparatively less investigated, as a result of their harder‐to‐control coordination number and environment, owing to the lack of ligand field stabilization, and of their affinity for water acting as a potential competing ligand thereby decreasing the dimensionality of the materials [6, 7, 8, 9]. While these aspects represent hurdles in the preparation of AE‐MOFs, these compounds remain quite appealing for the very presence of AE metal cations they are built on, as these show non/low toxicity and are abundant and affordable. For example, Mg‐MOF 74, also known as CPO‐27 [Mg_2_(2,5‐dobdc)] (2,5‐dobdcH_4_ = 2,5‐dihydroxyterephthalic acid, Figure 1), has shown remarkable CO_2_ adsorption capacity [10, 11, 12, 13, 14, 15], and [Mg(2,5‐dobdcH_2_)(H_2_O)_2_)](DMAc) (DMAc = *N*,*N*‐dimethylacetamide) has been found to be an efficient sensor for traces of water in organic solvents while the Ca(II), Sr(II) and Ba(II) analogues have been found to be highly emissive in the solid state [16, 17].

Recently, we have been interested in the synthesis of AE‐MOFs under ionothermal conditions in deep eutectic solvents (DESs) [18, 19, 20, 21, 22]. These media are combinations of two or more compounds that, at the eutectic ratio, feature a melting point lower than the one of the ideal mixture and of their individual components [23, 24, 25, 26, 27, 28, 29, 30, 31, 32]. They show non flammability, low/non toxicity and a capacity to solubilize both organic and inorganic species. In particular, the 1:2 mixture of choline chloride (ChCl) with 2‐imidazolidinone (ethylene urea, e‐urea, Figure 1) has been shown to be a solvent of interest for MOF preparation, as it impacts the crystalline morphology and textural properties of Mg‐MOF‐74 and allows the isolation of water sensitive Ca‐MOFs [19, 20]. It is worth noting that, in the reported Ca‐ and Sr‐MOFs prepared in this DES, the e‐urea molecule is systematically present as a ligand via coordination of its carbonyl group, stabilized by hydrogen bonding of the NH moieties [20, 21, 22]. While this has a strong structuring effect for the secondary building unit (SBU), this prevented activation of the materials toward exploitation of their potential porosity. It is also worth noting that ChCl, present in the DES, is absent from the structure of these MOFs. These observations led to the question of exploring urothermal rather than ionothermal conditions, consisting in performing the synthesis solely with the urea derivative as solvent [33, 34, 35]. Remarkably, the urothermal strategy has demonstrated its efficiency in allowing the preparation of a wide scope of MOFs based on transition metal and lanthanide cations. However, only a few examples of AE‐MOFs, a Ca‐based material and a series of Sr‐MOFs, have been reported [36, 37]. Thus, although promising, this approach remains underexplored for AE‐MOFs and we undertook in this work to investigate its potential. As solvent, 1,3‐dimethylimidazolidin‐2‐one (e‐murea, Figure 1) was chosen, since it is liquid at room temperature (m.p. = 8°C) and does not feature NH groups, preventing the formation of strong stabilizing hydrogen bonds upon coordination. Given the unique above‐mentioned properties of AE‐MOFs prepared from 2,5‐dobdcH_4_, it was selected as ligand for this study. Furthermore, preparation of AE‐MOFs under urothermal conditions in e‐murea was also explored with the 2,3‐dobdcH_4_ isomer (Figure 1) to evaluate the impact of the presence of two neighbouring hydroxyl groups on the coordination to AE metal cations and hence on the structure and properties of the resulting materials. A series of nine new AE‐MOFs is described with their structures determined by single‐crystal X‐ray diffraction and their optical properties characterized by absorption and emission spectroscopy.

## 2 Results and Discussion

**FIGURE 2:**
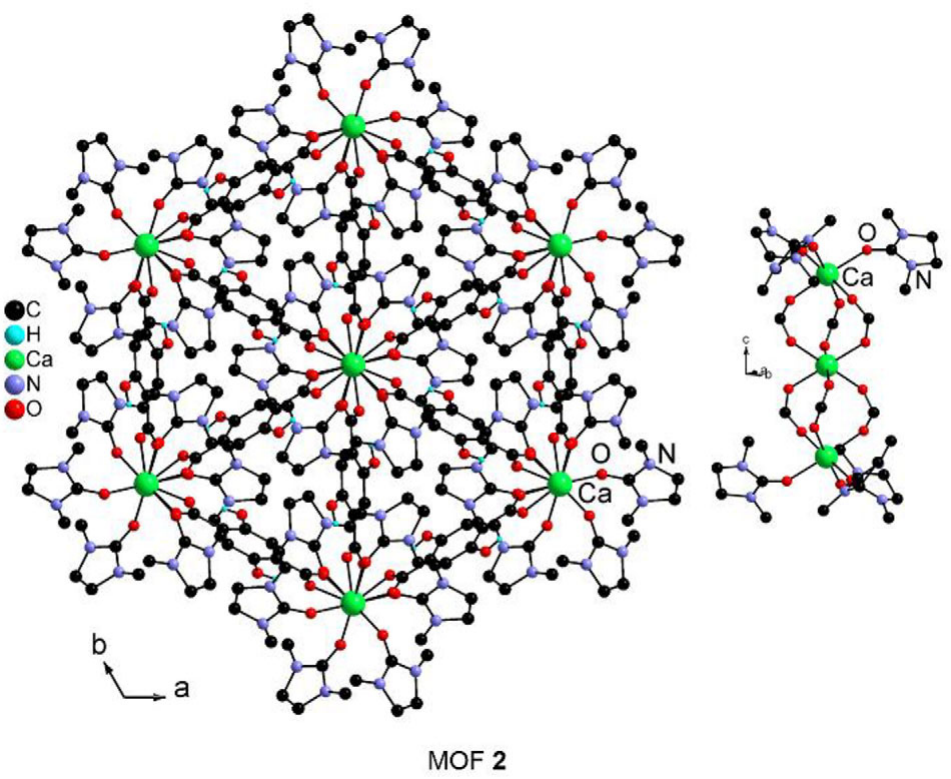
View along the *c* axis of **2** (left) and of the trinuclear SBU (right). CH hydrogen atoms have been omitted for clarity.

Upon heating a mixture of 2,5‐dobdcH_4_ with Mg(NO_3_)_2_·6H_2_O or Ca(NO_3_)_2_·4H_2_O in e‐murea, crystals of [M(2,5‐dobdcH_2_)(e‐murea)_2_] (M = Mg(II), MOF **1**; M = Ca(II), MOF **2**) were obtained. The two MOFs crystallize in the trigonal *R*‐3 space group and are isostructural (Table 3). They are built on two crystallographically independent metal cations both in an octahedral coordination environment, leading to a trinuclear SBU (Figure 2). The central cation is bound to six bridging carboxylate moieties, while the external one is ligated to three bridging carboxylates and three terminal e‐murea molecules. This leads to a 3D arrangement with triangular channels along the *c* axis, occupied by coordinated solvent molecules (Figure 2).

Such an organization is reminiscent of what has been reported for [Sr(bdc)(DMF)] [38] and [Sr(obdcH)(DMF)] [39] (bdcH_2_ = terephthalic acid; obdcH_3_ = 2‐hydroxyterephthalic acid; DMF = *N*,*N*‐dimethylformamide) also showing triangular channels albeit with every other channel being empty in these cases. It can be noted that the hydroxyl groups of the 2,5‐dobdcH_2_^2−^ anions remain protonated; they are intramolecularly hydrogen bonded to the carboxylate units (Table 3) and are not coordinated. It should also be emphasized that no traces of Mg‐MOF‐74 were observed under these urothermal conditions, unlike what has been reported under ionothermal conditions in reline and the 1:2 ChCl:e‐urea DES [19].

**FIGURE 3:**
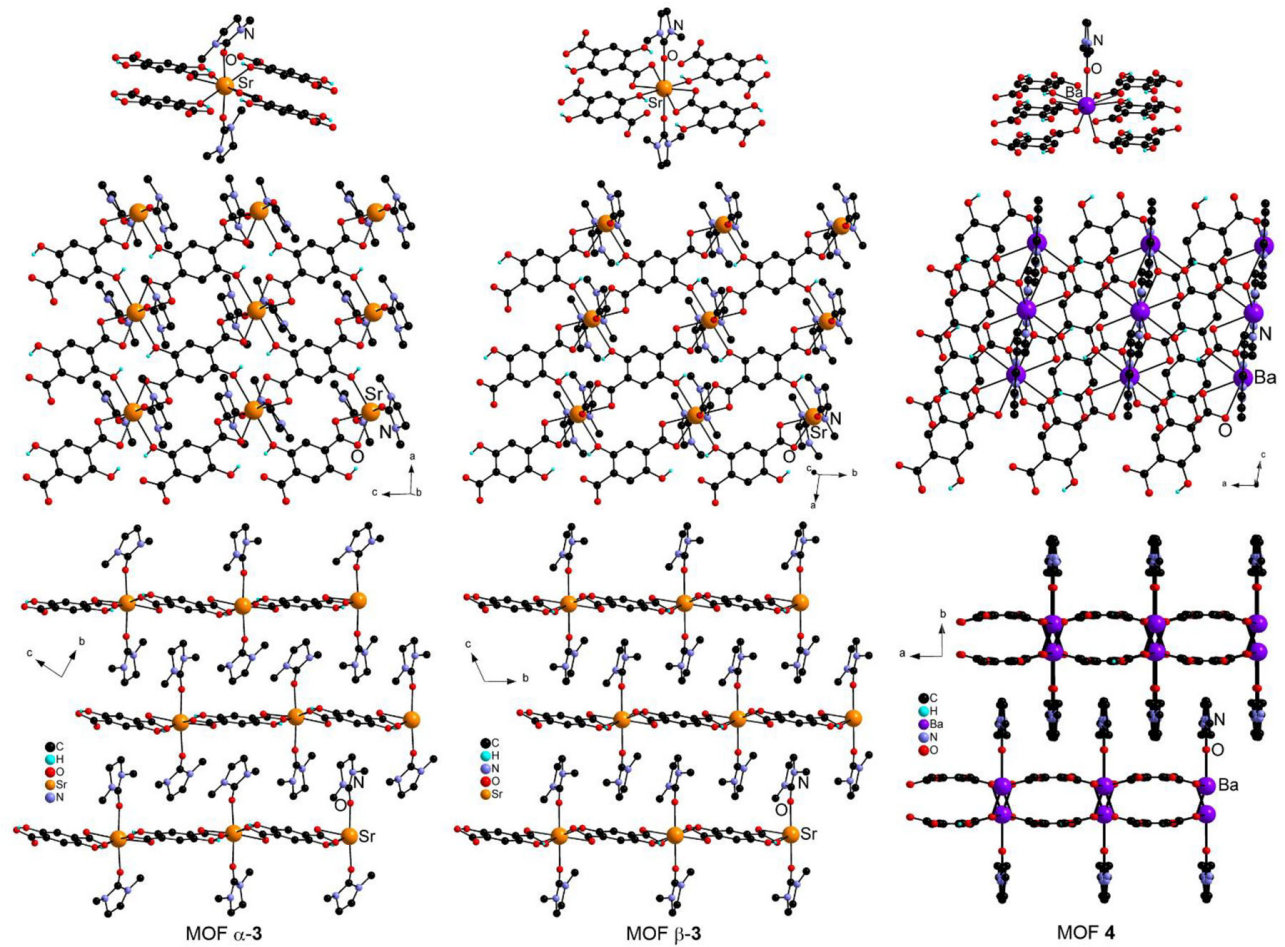
Views of the metal nodes (top), the 2D sheets (middle) and of their stacking (bottom) in α‐ and β‐**3** and **4**. Hydrogen atoms have been omitted for clarity. For the sake of clarity, hydrogen atoms have been omitted and only one position of the disordered e‐murea molecule has been presented in β‐**3**.

When the reaction was performed using Sr(NO_3_)_2_, two polymorphs formulated [Sr(2,5‐dobdcH_2_)(e‐murea)_2_], α‐ and β‐**3**, were isolated, both crystallizing in the triclinic *P*‐1 space group (Table 1). In these two phases, the Sr(II) cation is octa‐coordinated, bound to two chelating carboxylates, two hydroxyl groups and to two e‐murea molecules in the axial positions (Figure 3). This leads to the formation of 2D sheets that stack with interdigitated e‐murea. The asymmetric units of both structures comprise a single Sr(II) cation located in general position in α‐**3** and on an inversion center in β‐**3** (Figure SI25). Thus, it contains one independent coordinated e‐murea molecule in β‐**3** and two different e‐murea molecules bound to the metal cation in α‐**3**. While this impacts neither the coordination environment of the Sr(II) center nor the bond distances (Table 3) that are similar for the two structures, it results in different orientation of the e‐murea molecules between the two polymorphs (Figure 3). Unlike the Mg‐ and Ca‐MOFs described above, the hydroxyl groups are coordinating as reported for [Sr(2,5‐dobdcH_2_)(H_2_O)], CPO‐22 [40], and unlike [Sr(2,5‐dobdcH_2_)(DMAc)] [17].

**TABLE 1:** Selected distances and angles within the SBUs of MOFs **1–7** and geometric parameters (O‐H•••O distance and angle) of hydrogen bonds involving the hydroxyl groups.

	M‐O_carboxylate_ [Å]	M‐O_urea_ [Å]	M‐O_hydroxyl_ [Å]	O‐M‐O [°]	M‐M_withinSBUs_ [Å]	O‐H•••O [Å]	O‐H•••O [°]
**1**	2.0141(12)‐2.1320(10)	2.0718(12)		82.56(4)‐180	4.352	2.610(2)	145.8
**2**	2.2747(16)‐2.3670(14)	2.3068(17)		79.53(5)‐180	4.479(1)	2.572(2)	144.8
**α‐3**	2.5843(14)‐2.6959(15)	2.4685(16)‐2.4749(16)	2.742(1)‐2.797(1)	49.54(5)‐177.82(4)		2.542(2)‐2.580(2)	145.7‐146.4
**β‐3**	2.5907(19)‐2.701(5)	2.466(3)	2.765(6)	48.42(15)‐180		2.565(8)‐2.594(9)	143.2‐145.4
**4**	2.8092(17)‐2.9062(19)	2.9021(17)	2.628(3)	45.35(5)‐173.43(7)	4.8177(2)	2.597(3)	145.1
**α‐5**	2.386(4)‐2.396(4)	2.282(4)‐2.318(4)	2.444(4)‐2.490(4)	63.46(10)‐174.56(16)		2.467(5)‐2.469(5)	143.9‐144.2
**β‐5**	2.394(7)‐2.409(8)	2.276(8)‐2.409(8)	2.412(7)‐2.487(7)	63.0(2)‐176.8(4)		2.470(10)‐2.500(10)	144.0‐147.2
**6**	2.482(1)‐2.770(1)	2.482(1)	2.629(1)‐2.739(1)	48.08(3)‐160.87(4)	3.9144(3)	2.476(2)‐2.531(2)	145.3‐146.6
**7**	2.669(2)‐2.914(2)	2.643(1)	2.803(2)‐2.848(1)	45.68(4)‐161.11(4)	4.1267(2)	2.472(2)‐2.5101(2)	145.1‐146.2

So far, both phases were systematically obtained concomitantly, as demonstrated by X‐ray powder diffraction (Figure SI3), and could not be isolated individually in pure form, in spite of our numerous attempts to favour the formation of one polymorph over the other by modifying the concentration, temperature or duration of the reaction. It can be noted that the α‐ and β‐**3** polymorphs are the two phases that could be isolated and characterized by single crystal X‐ray diffraction. However, analysis of the batch by powder X‐ray diffraction indicates the presence of few peaks in addition to the ones expected from the combination of the two calculated patterns suggesting the presence of other minor phases (Figure SI3). Given that the elemental analysis results match the values expected for the common formula of α‐ and β‐**3**, potential additional phases are likely to be also polymorphs.

A 2D arrangement is also observed in [Ba(2,5‐dobdcH_2_)(e‐murea)], **4**, obtained upon reaction with Ba(NO_3_)_2_. It crystallizes in the monoclinic *I*2/*a* space group (Table 1). The Ba(II) cation is nine‐coordinated, bound to four carboxylates (two chelating and two bridging), two hydroxyl groups and one terminal e‐urea molecule. This coordination environment is reminiscent of what has been reported for [Ba(2,5‐dobdcH_2_)(DMAc)] [17]. However, here a 2D arrangement with a bilayer organization is observed (Figure 3). Along the *b* axis, these bilayers stack with interdigitated terminal coordinated solvent molecules, as for the two Sr polymorphs (Figure 3). It can be noted that this MOF differs also from the [Ba(2,5‐dobdcH_2_)] reported MOF, prepared from an imidazolium‐based ionic liquid [41].

Following the investigation of MOF formation using the 2,5‐dobdcH_4_, it appeared interesting to use the 2,3‐isomer (Figure 1) to evaluate the impact of the presence of two neighbouring hydroxyl groups on the SBU and hence on the MOFs. Furthermore, it is worth noting that, to the best of our knowledge, only one AE‐MOF has been previously reported with this latter ligand: [Ca(2,3‐dobdcH_2_)(H_2_O)] [42]. While, in our hands, the reaction of this ligand with Mg(NO_3_)_2_·6H_2_O in e‐murea has not led to the formation of crystalline material, MOFs could be isolated and characterized with Ca(II), Sr(II), and Ba(II).

**FIGURE 4:**
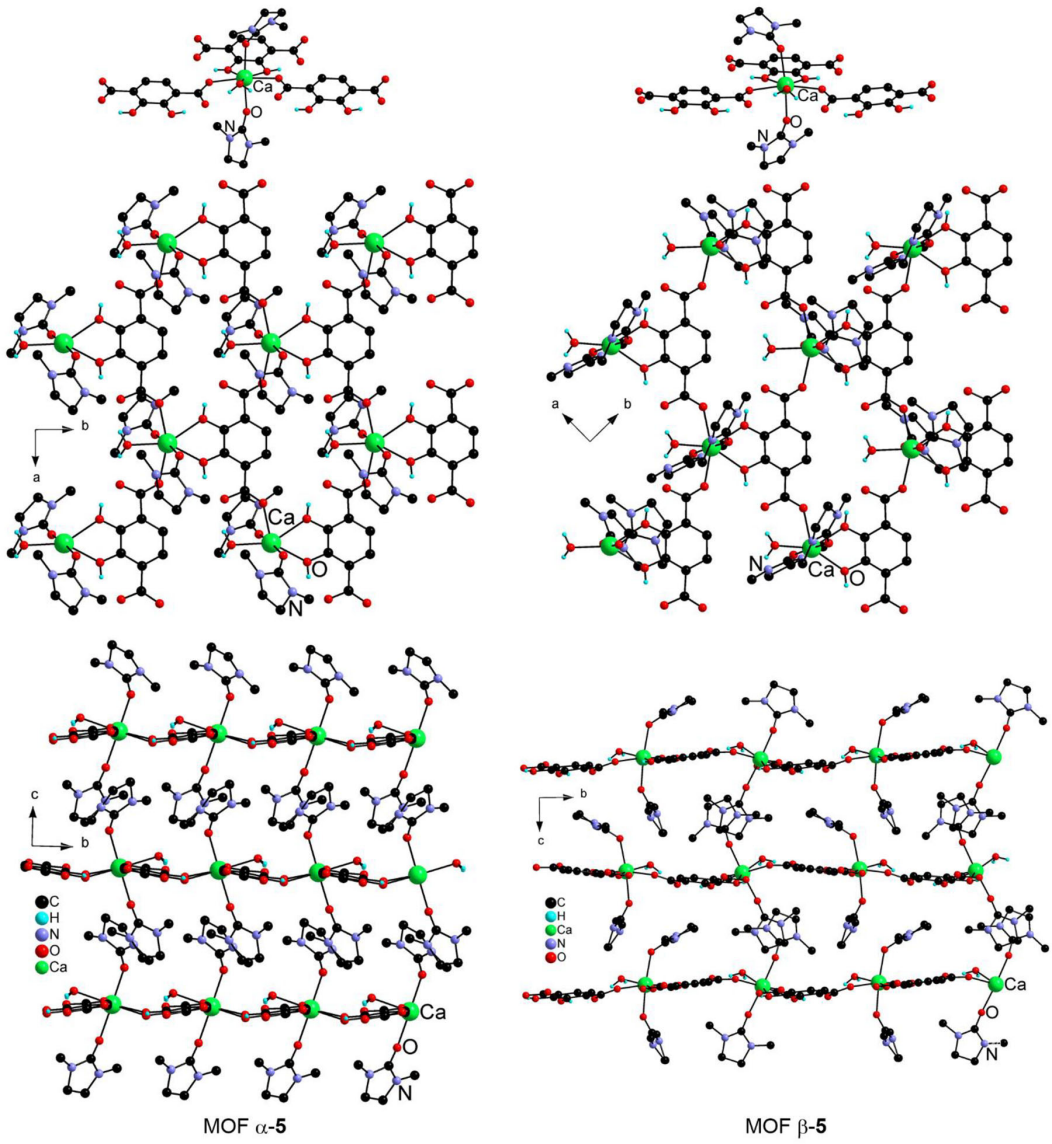
View of the metal node (top), 2D sheets (middle) and of their stacking (bottom) in α‐**5** (left) and β‐**5** (right). CH hydrogen atoms have been omitted for clarity.

Upon reaction of 2,3‐dobdcH_4_ with Ca(NO_3_)_2_·4H_2_O in e‐murea at 120°C for 3 days, two polymorphs formulated [Ca(2,3‐dobdcH_2_)(e‐murea)_2_(H_2_O)], α‐ and β‐**5**, formed and were characterized by single crystal X‐ray diffraction. Both polymorphs crystallize concomitantly as shown by powder X‐ray diffraction (Figure SI5) and could not be isolated individually in pure form, in spite of our attempts at modifying the reaction conditions. α‐ and β‐**5**, both crystallize in noncentrosymmetric space groups, *Cc* and *Pn* respectively (Table 2). They are built around a seven‐coordinated Ca(II) cation bound to two carboxylates, two hydroxyl groups in chelate fashion and two e‐urea and one water molecules. The water molecule may come from the starting metallic salt or from the e‐murea solvent, shown to contain 0.169% of water by Karl Fischer titration. Bridging by the 2,3‐dobdcH_2_^2−^ anion leads to the formation of 2D layers that stack along the *c* axis (Figure 4). The two phases differ in the relative orientation of the layers and of the coordinated e‐urea molecules. This results from the different space groups as well as the fact that the asymmetric unit of α‐**5** comprises one Ca(II) cation, one ligand, two e‐murea molecules and one water, whereas β‐**5** is based on twice these components (Figure SI25). Although the coordination environment of the different Ca(II) cations and the bond distances are similar, the position of the bound e‐murea molecule varies leading to these differing relative orientations when comparing the two structures. It is interesting to highlight that, in the Ca‐MOFs described here, while the hydroxyl groups systematically form intramolecular hydrogen bonds with neighbouring carboxylates, they are coordinating in α‐ and β‐**5** and not in **2**.

**TABLE 2:** Photophysical data for MOFs **1–7** at room temperature and 77 K.

MOF	*λ*_em_ [nm]^a^	τ [ns]^b^	*λ*_em_ [nm]^c^	τ [ns]^d^	ϕ^a^
**1**	495	3.9; 9.4^e^	495	9.3; 14.4^e^	0.84
**2**	500	8.8^e^	500	10.5^e^	0.62
**3**	565	5.8; 10.0^f^	565	5.9; 11.3^f^	0.45
**4**	555	2.6; 7.2^g^ 3.3; 7.2^h^	585	4.2; 9.2^g^ 4.3; 8.7^h^	0.49
**5**	435		445		0.55
**6**	450		455		0.70
**7**	450		455		0.45

^a^ *λ*_ex_ = 360 nm, T = 293 K;

^b^ T = 293 K;

^c^ *λ*_ex_ = 360 nm, T = 77 K;

^d^ T = 77 K;

^e^ Monitored at 484 nm;

^f^ Monitored at 570 nm;

^g^ Monitored at 500 nm;

^h^ Monitored at 600 nm.

**FIGURE 5:**
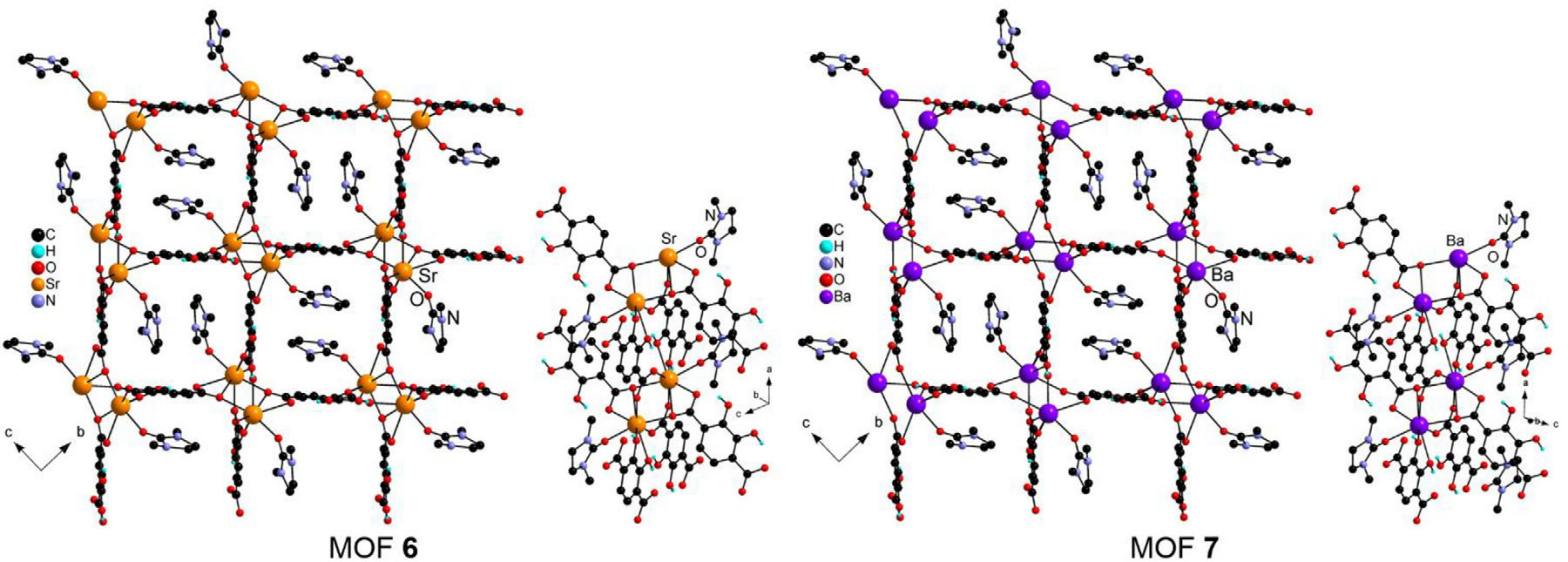
View along the *a* axis (left) and of the SBU (right) of the structure of Sr‐MOF **6** (top) and Ba‐MOF **7** (bottom). CH hydrogen atoms have been omitted for clarity.

Coordination in chelate fashion of the dihydroxyl moiety is also observed in the two MOFs formulated [M(2,3‐dobdcH_2_)(e‐murea)], **6** (M = Sr(II)) and **7** (M = Ba(II)). **6** and **7** crystallize respectively in the *P*2_1_/*c* and *P*2_1_/*n* space groups (Table 2) and feature analogous structures with a underlying framework characterized by the {4^3^.6^5^.8^2^}{4^7^.6^3^} point symbol. The arrangement in **6** is shown in Figure 5 as a representative example. The metal center is eight‐coordinated, bound to four bridging carboxylate units, one chelating dihydroxyl group and a terminal e‐murea molecule, yielding a binuclear unit that forms 1D pillars (Figure 5). Bridging of these pillars leads to the formation of a 3D arrangement with channels occupied by coordinated solvent molecules along the *a* axis (Figure 5).

One can note that, going from Mg(II) to Ba(II), an increase in the coordination number is observed with both ligands. It goes from 6 for MOFs **1** and **2** to 8 for α‐ and β‐**3** and 9 for **4**, incorporating the 2,5‐dobdcH_2_^2−^ ligand and from 7 for α‐ and β‐**5** to 8 for MOFs **6** and **7** based on the 2,3‐dobdcH_2_^2−^ derivative. This is consistent with the increase in ionic radius when going down the column of alkaline earth metals and is reflected in the M‐O coordination distance increase observed in the crystal structures of the MOFs described herein (Table 3). One salient difference between the two series of MOFs is that with 2,3‐dobdcH_2_^2−^ derivative, chelation is observed, whereas coordination by the two hydroxyl groups is only present with the Sr(II) and Ba(II) MOFs **3** and **4**, based on the 2,5‐dobdcH_2_^2−^ derivative.

**TABLE 3:** Crystallographic data for MOFs **1–4**.

	1	2	α‐3	β‐3	4
Formula	C_18_H_24_MgN_4_O_8_	C_18_H_24_CaN_4_O_8_	C_18_H_24_N_4_O_8_Sr	C_18_H_24_N_4_O_8_Sr	C_13_H_14_BaN_2_O_7_
FW	448.72	464.49	512.03	512.03	447.60
Crystal system	Trigonal	Trigonal	Triclinic	Triclinic	Monoclinic
Space group	*R*‐3	*R*‐3	*P*‐1	*P*‐1	*I*2/*a*
*a* [Å]	17.9338(5)	18.644(4)	7.0161(2)	7.0156(5)	7.4316(3)
*b* [Å]	17.9338(5)	18.644(4)	10.0600(4)	8.6081(6)	23.3936(10)
*c* [Å]	15.9048(7)	16.110(4)	15.0601(6)	9.4939(7)	8.7448(4)
*α* [°]	90	90	84.142(2)	112.743(3)	90
*β* [°]	90	90	89.1110(10)	98.117(3)	98.184(2)
*γ* [°]	120	120	76.1350(10)	97.338(3)	90
*V* [Å^3^]	4430.0(3)	4850(2)	1026.57(7)	512.93(6)	1504.82(11)
*Z*	9	9	2	1	4
*λ* [Å]	0.71073	0.71073	0.71073	0.71073	0.71073
*T* [K]	120(2)	120(2)	120(2)	120(2)	120(2)
*μ* [mm^−1^]	0.147	0.343	2.681	2.683	2.678
Refls. coll.	15960	16191	38233	11173	25448
Ind. refls. (R_int_)	2385 (0.0605)	2556 (0.0231)	6013 (0.0492)	2463 (0.0437)	2206 (0.0476)
*R_1_* (I>2 σ(I))^a^	0.0377	0.0476	0.0356	0.0404	0.0240
*wR_2_* (I>2σ(I))^a^	0.0842	0.1364	0.0786	0.0967	0.0509
*R_1_* (all data)^a^	0.0590	0.0507	0.0473	0.0506	0.0253
*wR_2_* (all data)^a^	0.0946	0.1393	0.0855	0.1027	0.0512
*GOF*	1.020	1.089	1.058	1.042	1.315

^a^ *R*_1_ = ∑ll*F_o_* l‐l*F_c_* ll/∑l*F_o_* l; *wR*_2_ = [∑*w*(*F_o_* ^2^‐*F_c_* ^2^)^2^/∑w*F_o_* ^4^]^1/2^.

The MOFs were characterized by X‐ray powder diffraction to confirm the presence of a single phase in the batch (Figures SI1–SI7). For **1**, **2**, **4**, **6**, and **7**, a good match is indeed observed between the pattern calculated from single‐crystal data and the experimental one. The purity of the materials is further confirmed by elemental analysis. Infra‐red spectroscopy indicated the absence of remaining starting ligand (Figures SI8–SI14) as demonstrated in particular in the area between 1500 and 1700 cm^−1^ showing the emergence of the ν_as_(COO^−^) mode of the carboxylate upon coordination to the AE metal cation and formation of the MOFs, concomitant with the disappearance of the ν(C═O) stretching mode [43]. As mentioned above, the X‐ray diffraction pattern indicates the presence of few peaks in addition to the ones expected for a combination of the α‐ and β‐**3** phases, suggesting the presence of other potential crystalline systems (Figure SI3). Interestingly, the elemental analysis results match the formula for the α and β polymorphs, indicating that other potential MOFs most likely have the same composition as these and are likely to be other polymorphs. For **5**, the X‐ray diffraction pattern matches a combination of the calculated diagrams for α‐ and β‐**5** (Figure SI5). In light of the similarities in coordination environment and organization within each pair (α‐**3** and β‐**3**, on one hand, and α‐**5** and β‐**5**, on the other hand), each system was subsequently analyzed as a pair combining both polymorphs, named **3** and **5** respectively.

To explore the possibility to thermally activate the materials to access their potential porosity, thermogravimetric analysis was performed (Figures SI15–SI21). For the 3D MOF **1**, two consecutive weight losses between 200 and 300°C corresponding to 34 and 31% are observed exceeding the expected 50% of e‐murea molecules. Furthermore, since only small amount of this product could be isolated in light of the low yield, the porosity of this material could not be further studied. For **2**, no weight loss is observed up to 250°C, where a 65% weight loss, accounting to more than the 49% expected for the two e‐murea molecules, is observed therefore suggesting decomposition of the materials. For the 2D MOFs **3–6**, a similar behaviour is also observed. Finally, the 3D Ba‐MOF **7** also shows a sharp weight loss of ca 45% at 300°C corresponding to more than the mass of e‐murea (26%), hence preventing further exploration of thermal activation for this material. It is interesting to note that such an issue in activating AE‐MOFs prepared under iono‐ or urothermal conditions in e‐urea based solvents has also been reported [19, 20, 21, 22, 37]. For these latter materials, the key role of hydrogen bonds between the NH moieties and the carboxylate groups was hypothesized as a key factor in the difficulty in activating the MOFs, as the removal of e‐urea was envisioned as a structurally destabilizing factor. However, here, regardless of the nature of the metal cations, coordination of e‐murea is observed in terminal fashion for all materials and no strong hydrogen bonding is observed with the carboxylate moieties. This suggests that the coordination mode of the urea derivative may not be the sole factor. Given that numerous porous AE‐MOFs have been reported in the literature [6, 7, 8, 9, 10, 11, 12, 13, 14, 15, 19], the nature of the metal cation cannot be invoked. One hypothesis for the challenging activation of the compounds described herein may be the generation of decomposition product(s) from the urea species upon heating that could lead to destabilization of the porous architecture. In order to circumvent this issue, we have attempted to perform post‐synthetic solvent exchange to remove the e‐murea before thermal activation. Unfortunately, this also proved unsuccessful.

**FIGURE 6:**
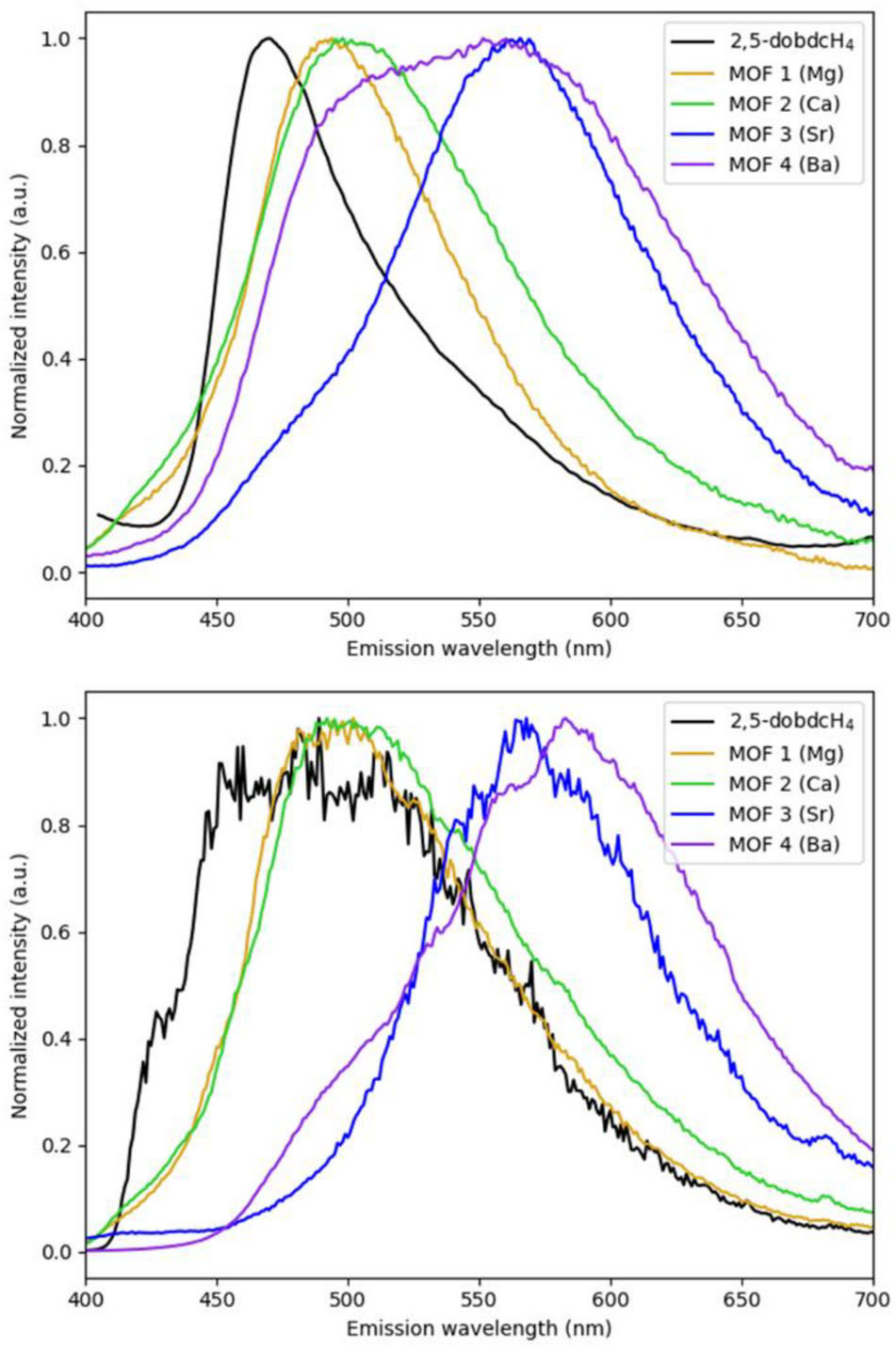
Solid‐state emission spectra of MOFs **1–4** based on the 2,5‐dobdcH_2_^2−^ ligand, upon excitation at 360 nm at 293 K (top) and 77 K (bottom).

In light of the reported interesting photophysical properties of MOFs based on 2,5‐dobdcH_2_^2−^ [16, 17], the luminescence of **1–7** in the crystalline state was investigated (Figure 6 and Table 2) at room temperature. For Mg‐MOF **1**, an intense emission at 495 nm (ϕ = 0.84) upon excitation at 360 nm, the absorption maximum. This emission is slightly red‐shifted for the Ca isostructural analogue **2** (*λ*_em_ = 500 nm, ϕ = 0.62). The red‐shift increases and the quantum yield decreases upon moving to heavier AE cations, with Sr‐ and Ba‐based **3** and **4** showing emission at 565 and 555 nm, respectively. It is worth noting that, in the case of **4**, a shoulder at 500 nm is observed, in agreement with what has been reported for [Ba(2,5‐dobdcH_2_)(DMAc)] [17]. This has been rationalized in the literature by the fact that emission can proceed from two excited states in thermal equilibrium [17].

First, the so‐called normal state is attributed to a higher lying excited state formed after vibrational relaxation from S_1_ of the bridging 2,5‐dobdcH_2_^2−^ ligand. The second one derives from the earlier upon excited state intramolecular proton transfer (ESIPT) [44] from the hydroxyl group to the carboxylate. The ESIPT is expected to be favoured with heavier AE cations as the larger ionic radius leads to longer metal‐carboxylate bond distances, thus limiting the negative impact of coordination of this moiety on the proton transfer [17]. ESIPT is also assisted by coordination of the hydroxyl groups.

Therefore, it can be hypothesized that this phenomenon is not favoured in **1** and **2** incorporating lighter Mg(II) and Ca(II) and for which the hydroxyl groups are not coordinated, hence leading to the observation of one emission (Figure 6 top). In contrast, it can be rationalized that the observed red‐shift in emission for **3** and **4** and the presence of a shoulder (Figure 6 top) are related to two concurring factors, the nature of the AE cations leading to lengthening of the metal‐carboxylate bond distances (Table 1) as well as coordination of the hydroxyl in these MOFs, favouring the coexistence of the two types of emission. To confirm the presence of two emission phenomena in thermal equilibrium, the solid‐state luminescence was measured at 77 K (Figure 6 bottom). While the emission of **1** and **2** remained similar to what is observed at room temperature, the shoulder at higher energy decreased for **3** and **4** (Table 2). This suggests that in the latter material the contribution from the excited state resulting from the ESIPT is predominant at lower temperature, consistently with what has been described for [Ba(2,5‐dobdcH_2_)(DMAc)] [17]. It is worth noting that, besides the nature of the metal cation, what differentiates **1** and **2** from **3** and **4** is the dimensionality of the MOFs. A reduction of dimensionality is indeed observed going from Mg(II) and Ca(II) which yield 3D architectures to Sr(II) and Ba(II) affording 2D materials. However, the dimensionality of the frameworks does not seem at stake for the observed dual emission, given that such phenomenon has been observed for analogous Sr(II) and Ba(II) systems with a 3D structural arrangement [17]. It can also be noted that the excited state lifetimes for the four MOFs is in the range of few ns (Table 2), consistent with reported analogous systems [17].

**FIGURE 7:**
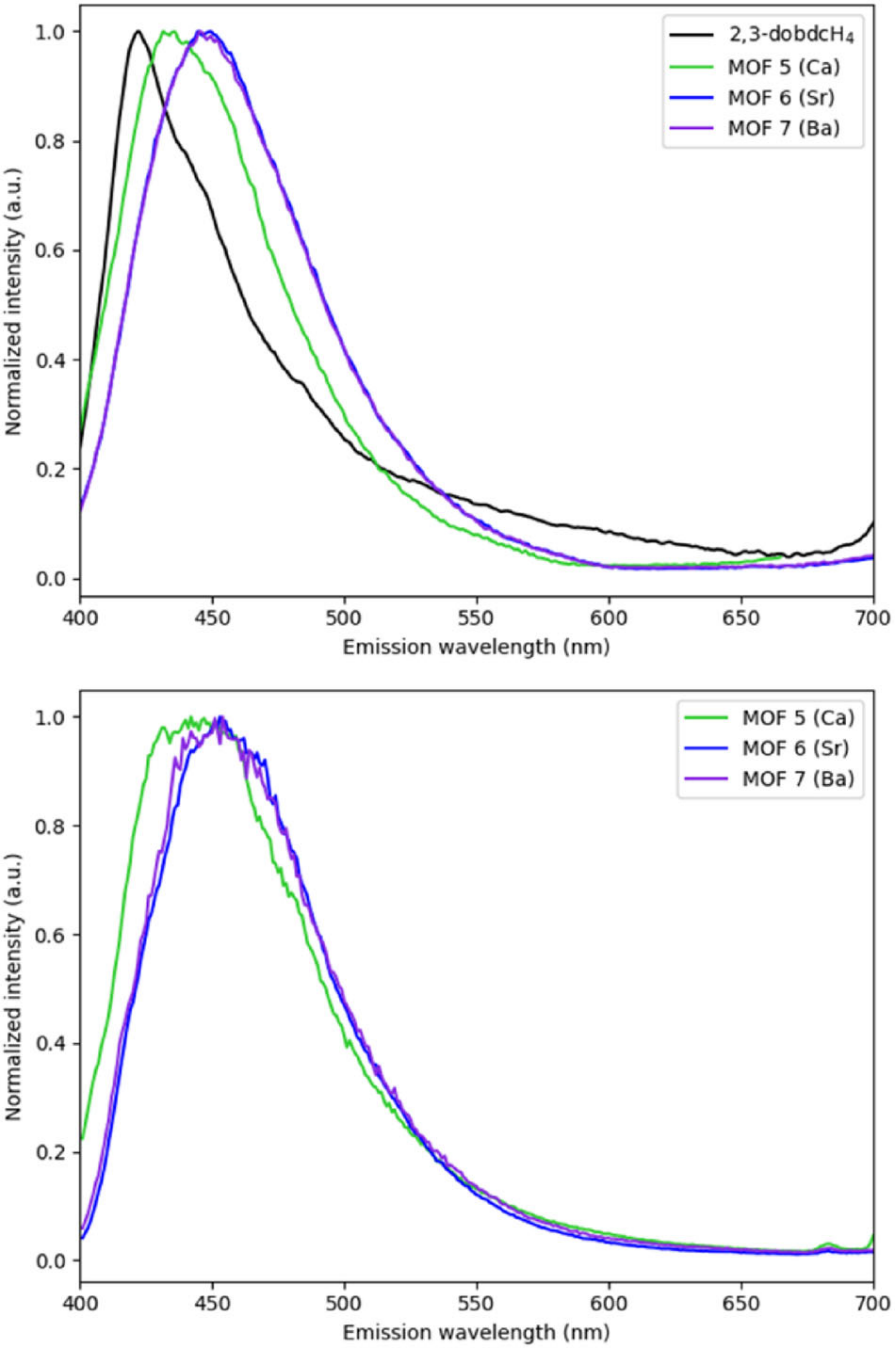
Solid‐state emission spectra of **5–7** based on the 2,3‐dobdcH_2_^2−^ ligand, upon excitation at 360 nm at 293 K (top) and 77 K (bottom).

Interestingly such strong metal‐ and temperature‐dependence of the emission is not observed for MOFs based on the 2,3‐dobdcH_2_^2−^ analogue (Figure 7). A 15‐nm red shift is detected between the Ca‐based system **5**, and the Sr(II) and Ba(II) analogues **6** and **7** (Figure 7 and Table 2). It can be hypothesized that the chelate coordinating mode of the hydroxyl groups in these MOFs disfavours the ESIPT, thereby leading to the observed smaller red shift and the absence of a structured emission for the Ba(II) system **7**.

For **1–7**, the quantum yield at room temperature exceeds 0.45 (Table 2), a rather remarkable result when compared with other reported emissive AE‐MOFs [17, 22, 37, 45, 46]. In particular, it can be noted that for other MOFs based on the 2,5‐dobdcH_2_^2−^ ligand, the quantum yield was reported to range between 0.26 and 0.54 depending on the nature of the alkaline earth metal cations [17].

## 3 Conclusion

In this study, the urothermal approach in e‐murea was employed for the successful preparation of a series of nine AE‐MOFs with two different dihydroxy terephthalic acid ligands. The compounds were characterized by single‐crystal and powder X‐ray diffraction as well as elemental analysis and infra‐red spectroscopy to confirm their purity. In all materials, coordination of the solvent molecule in terminal fashion is observed in contrast with what has been previously reported under iono‐ and uro‐thermal conditions in solvents based on e‐urea featuring NH moieties prone to hydrogen bonding to neighbouring carboxylate groups. With the 2,5‐dobdcH_2_^2−^ ligand, two isostructural 3D MOFs were obtained with Mg(II) and Ca(II), **1** and **2**, whereas 2D architectures were isolated with Sr(II) and Ba(II), **3** and **4**. Beyond their dimensionality, they also differ in the coordination of the hydroxyl groups in **3** and **4**. Binding of these groups in a chelate fashion is consistently observed in AE‐MOFs **5–7** prepared with the 2,3‐dobdcH_2_^2−^ ligand. While the terminal mode of coordination of the solvent suggested potential activation of the materials, thermogravimetric analysis indicated decomposition concomitant with the solvent loss. While the porosity of these MOFs could therefore not be explored, their photophysical properties were investigated, indicating strong luminescence with quantum yields ranging between 0.45 and 0.84, remarkable for this type of compounds. Interestingly for MOFs based on the 2,5‐dobdcH_2_^2−^ ligand, a dependence on the nature of the alkaline earth metal cation was observed, expressed in the presence of two different emission processes for the heavier Sr(II) and Ba(II) systems. Based on literature precedent, this phenomenon was rationalized by ESIPT leading to luminescence from another emitting state in addition to relaxation from the S_1_ state. The two processes are in thermal equilibrium as confirmed by investigation of the solid‐state luminescence at 77K showing a predominant contribution of emission resulting from the ESIPT‐induced state at lower temperature. Interestingly, for the AE‐MOFs based on the 2,3‐dobdcH_2_^2−^ ligand, such metal and temperature dependence is not observed. The foregoing results demonstrate the viability of e‐murea to be employed for the urothermal preparation of MOFs. Future investigation will center on the extension of the use of this solvent for the synthesis of MOFs based on other metal cations and ligands aiming at the development of novel porous/luminescent materials and will be published in due course. The development of porous systems featuring ESIPT based emission pathways is currently being pursued to take advantage of the synergy of the two properties toward sensing or responsive materials, as recently reported in the literature [47, 48, 49, 50].

## 4 Experimental Section

### 4.1 Synthesis

All reagents were obtained from commercial sources and used as received: 1,3‐dimethylimidazolidin‐2‐one (98%, Fluorochem), 2,5‐dihydroxyterephthalic acid (97%, Abcr), 2,3‐dihydroxyterephthalic acid (97%, BLD pharm), Mg(NO_3_)_2_·6H_2_O (99%, Aldrich), Ca(NO_3_)_2_·4H_2_O (97%, Alfa‐Aesar), Sr(NO_3_)_2_ (99%, Strem Chemicals), and Ba(NO_3_)_2_ (99%, Fluka)

#### 4.1.1 General MOF Synthesis Procedure

The solid reagents were added to a vial, followed by addition of the solvent. The vial was then heated in a dry bath. After completion, the reaction was filtered and washed multiple times with ethanol. The remaining solid was air‐dried.

#### 4.1.2 MOF 1 [Mg(2,5‐dobdcH_2_)(e‐murea)_2_]

2,5‐dihydroxyterephthalic acid (0.040 g, 0.2 mmol) and Mg(NO_3_)_2_·6H_2_O (0.102 g, 0.4 mmol) were added to a 20 mL vial before addition of 10 mL of e‐murea. The vial was then heated at 80°C for 3 days. The reaction was treated according to the general procedure (0.0073 g, 8.8%). Elemental analysis (CHN) for C_18_H_24_MgN_4_O_8_; calculated: C, 48.18; H, 5.39; N, 12.49; found: C, 48.01; H, 5.39; N, 12.48.

#### 4.1.3 MOF 2 [Ca(2,5‐dobdcH_2_)(e‐murea)_2_]

2,5‐dihydroxyterephthalic acid (0.079 g, 0.4 mmol) and Ca(NO_3_)_2_·4H_2_O (0.189 g, 0.8 mmol) were added to a 20 mL vial before addition of 10 mL of e‐murea. The vial was then heated at 120°C for 3 days. The reaction was treated according to the general procedure (0.0772 g, 41.6%). Elemental analysis (CHN) for C_18_H_24_CaN_4_O_8_; calculated: C, 46.55; H, 5.21; N, 12.06; found: C, 46.24; H, 5.20; N, 12.02.

#### 4.1.4 MOF 3 [Sr(2,5‐dobdcH_2_)(e‐murea)_2_]

2,5‐dihydroxyterephthalic acid (0.079 g, 0.4 mmol) and Sr(NO_3_)_2_ (0.169 g, 0.8 mmol) were added to a 20 mL vial before addition of 10 mL of e‐murea. The vial was then heated at 120°C for 3 days. The reaction was treated according to the general procedure to yield a combination of α‐**3** and β‐**3** (0.0658 g, 32.1%). Elemental analysis (CHN) for C_18_H_24_N_4_O_8_Sr; calculated: C, 42.22; H, 4.72; N, 10.94; found: C, 42.08; H, 4.78; N, 11.15.

#### 4.1.5 MOF4 [Ba(2,5‐dobdcH_2_)(e‐murea)]

2,5‐dihydroxyterephthalic acid (0.040 g, 0.2 mmol) and Ba(NO_3_)_2_ (0.105 g, 0.4 mmol) were added to a 20 mL vial before addition of 10 mL of e‐murea. The vial was then heated at 120°C for 3 days. The reaction was treated according to the general procedure (0.0665 g, 74.3%). Elemental analysis (CHN) for C_13_H_14_BaN_2_O_7_; calculated: C, 34.89; H, 3.15; N, 6.26; found: C, 34.80; H, 3.22; N, 6.46.

#### 4.1.6 MOF 5 [Ca(2,3‐dobdcH_2_)(e‐murea)_2_(H_2_O)]

2,3‐dihydroxyterephthalic acid (0.079 g, 0.4 mmol) and Ca(NO_3_)_2_·4H_2_O (0.189 g, 0.8 mmol) were added to a 20 mL vial before addition of 10 mL of e‐murea. The vial was then heated at 120°C for 3 days. The reaction was treated according to the general procedure to yield a combination of α‐**5** and β‐**5** (0.0247 g, 12.8%). Elemental analysis (CHN) for C_18_H_26_CaN_4_O_9_; calculated: C, 44.81; H, 5.43; N, 11.61; found: C, 44.70; H, 5.45; N, 11.80.

#### 4.1.7 MOF 6 [Sr(2,3‐dobdcH_2_)(e‐murea)]

2,3‐dihydroxyterephthalic acid (0.079 g, 0.4 mmol) and Sr(NO_3_)_2_ (0.169 g, 0.8 mmol) were added to a 20 mL vial before addition of 10 mL of e‐murea. The vial was then heated at 120°C for 3 days. The reaction was treated according to the general procedure (0.0287 g, 15.5%). Elemental analysis (CHN) for C_13_H_14_N_2_O_7_Sr; calculated: C, 39.24; H, 3.55; N, 7.04; found: C, 39.02; H, 3.64; N, 7.40.

#### 4.1.8 MOF 7 [Ba(2,3‐dobdcH_2_)(e‐murea)]

2,3‐dihydroxyterephthalic acid (0.040 g, 0.2 mmol) and Ba(NO_3_)_2_ (0.105 g, 0.4 mmol) were added to a 20 mL vial before addition of 10 mL of e‐murea. The vial was then heated at 120°C for 3 days. The reaction was treated according to the general procedure (0.0378 g, 42.2%). Elemental analysis (CHN) for C_13_H_14_BaN_2_O_7_; calculated: C, 34.89; H, 3.15; N, 6.26; found: C, 34.86; H, 3.23; N, 6.53.

### 4.2 X‐ray Diffraction

Data [51] (Tables 3, 4) were collected on a Bruker PHOTON‐III DUO CPAD diffractometer equipped with an Oxford Cryosystem liquid N_2_ device, using Mo‐Kα radiation (*λ* = 0.71073 Å) at 120 K. The structures were solved using the program SHELXT‐2018 [52]. The refinement and all further calculations were carried out using SHELXL‐2018 [53]. The H‐atoms were included in calculated positions and treated as riding atoms using SHELXL default parameters. The non‐H atoms were refined anisotropically, using weighted full‐matrix least‐squares on F^2^. A semi‐empirical absorption correction was applied using SADABS in APEX4 [54]. In the structure of β‐**3**, a e‐murea molecule is disordered over two positions that have been modelled using the PART command of SHELXL [53].

**TABLE 4:** Crystallographic data for MOFs **5–7**.

	α‐5	β‐5	6	7
Formula	C_18_H_26_CaN_4_O_9_	C_36_H_52_Ca_2_N_8_O_18_	C_13_H_14_N_2_O_7_Sr	C_13_H_14_BaN_2_O_7_
FW	482.51	965.01	397.88	447.60
Crystal system	Monoclinic	Monoclinic	Monoclinic	Monoclinic
Space group	*Cc*	*Pn*	*P*2_1_/*c*	*P*2_1_/*n*
*a* [Å]	11.7888(5)	8.3554(11)	8.4584(2)	8.6728(3)
*b* [Å]	11.7972(6)	16.544(2)	13.4594(3)	13.6649(6)
*c* [Å]	16.8082(8)	16.151(2)	13.3890(3)	13.7905(6)
*α* [°]	90	90	90	90
*β* [°]	104.736(2)	92.302(4)	108.3810(10)	108.2430(10)
*γ* [°]	90	90	90	90
*V* [Å^3^]	2260.71(19)	2230.8(5)	1446.50(6)	1552.21(11)
*Z*	4	2	4	4
*λ* [Å]	0.71073	0.71073	0.71073	0.71073
*T* [K]	120(2)	120(2)	120(2)	120(2)
*μ* [mm^−1^]	0.333	0.338	3.768	2.596
Refls. coll.	20348	30438	31913	31045
Ind. refls. (R_int_)	5758 (0.0419)	9987 (0.0605)	4628 (0.0431)	4511 (0.0359)
*R_1_* (I>2 σ(I))^a^	0.0567	0.1162	0.0242	0.0200
*wR_2_* (I>2σ(I))^a^	0.1493	0.3321	0.0455	0.0451
*R_1_* (all data)^a^	0.0607	0.1318	0.0342	0.0254
*wR_2_* (all data)^a^	0.1523	0.3471	0.0488	0.0475
*GOF*	1.116	1.439	1.051	1.080

^a^ *R*_1_ = ∑ll*F_o_* l‐l*F_c_* ll/∑l*F_o_* l; *wR*_2_ = [∑*w*(*F_o_* ^2^‐*F_c_* ^2^)^2^/∑w*F_o_* ^4^]^1/2^.

Powder X‐ray diffraction patterns were recorded at 293 K on a Bruker D8 diffractometer using monochromatic Cu‐Kα radiation with a scanning range between 4 and 40° using a scan step of 1.17° min^−1^. The simulated diagrams were generated with the Mercury software [55] based on the single‐crystal data collected.

### 4.3 Thermo‐Gravimetric Analysis

The thermal stability of the samples was determined on a PerkinElmer thermogravimetric analyzer TGA 4000 under N_2_ flow of 20 mL.min^−1^ and at a heating rate of 5°C.min^−1^ up to 800°C.

### 4.4 Optical Properties

Emission spectra at room temperature were collected on a PerkinElmer LS55 fluorescence spectrometer. Steady‐state emission spectra at 77K were recorded on a Horiba Jobin–Yvon IBH FL‐322 Fluorolog 3 spectrometer equipped with a 450 W xenon arc lamp, double‐grating excitation, and emission monochromators (2.1 nm mm−1 dispersion; 1200 grooves per mm) and a Hamamatsu R13456 red‐sensitive Peltier‐cooled PMT detector. Emission and excitation spectra were corrected for source intensity (lamp and grating). Quantum yields were determined on a Hamamatsu Quantaurus QY absolute PL quantum yield spectrometer C11347. Luminescnece lifetimes were measured on a PicoQuant FT300 spectrometer.

### 4.5 Elemental Analysis

Elemental analyses (CHN) were performed at the Service Commun d'Analyses of the University of Strasbourg, in duplicate, employing ThermoFisher FLASH 2000 equipment, whereas the reported values for CHN were taken as the average of two measurements.

## Conflicts of Interest

The authors declare no conflicts of interest.

## Supporting information



Powder X‐ray diffraction pattern, thermogravimetric analysis, infra‐red and excitation spectra, and crystallographic data of MOFs **1–7**.
