# Supplementary material for: Urothermal Synthesis of Luminescent Alkaline Earth MOFs
Source: Chemistry. 2026 Apr 18;32(25):e70996. doi: 10.1002/chem.70996 (PMC13331594; doi:10.1002/chem.70996)
Supplement: Supplementary file 1 — Powder X‐ray diffraction pattern, thermogravimetric analysis, infra‐red and excitation spectra, and crystallographic data of MOFs 1–7. [file CHEM-32-e70996-s001.docx]

Supporting Information

**Urothermal Synthesis of Luminescent Alkaline Earth MOFs**

Michael Teixeira, and Stéphane A. Baudron*

*Université de Strasbourg, CNRS, CMC UMR 7140, 4 rue Blaise Pascal, F-67000 Strasbourg, France. E-mail :* [*sbaudron@unistra.fr*](mailto:sbaudron@unistra.fr)

**Powder X-ray diffraction (PXRD)**


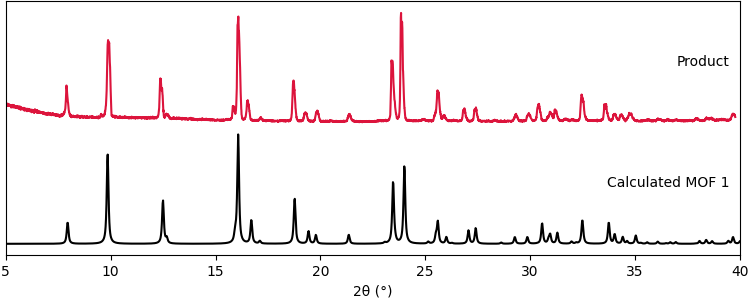


**Figure SI1.** Comparison of the PXRD diffractogram of a batch of Mg-MOF **1** (red, top) with the diagram calculated from single-crystal data (black, bottom).


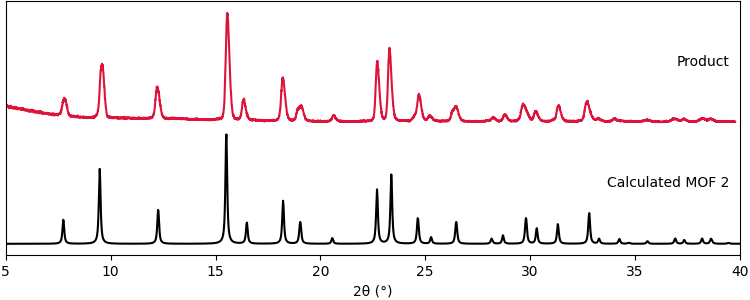


**Figure SI2.** Comparison of the PXRD diffractogram of a batch of Ca-MOF **2** (red, top) with the diagram calculated from single-crystal data (black, bottom).


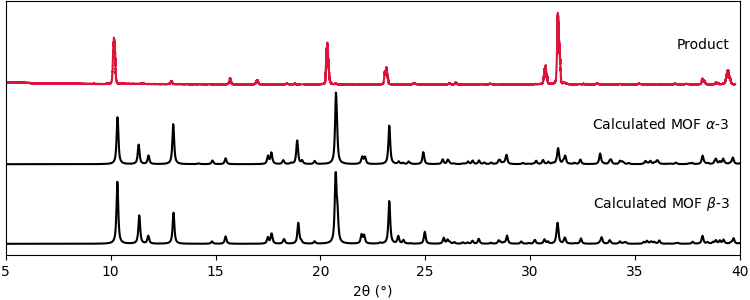


**Figure SI3.** Comparison of the PXRD diffractogram of a batch of Sr-MOF **3** (red, top) with the diagram calculated from single-crystal data for α and β-**3** (black, bottom).


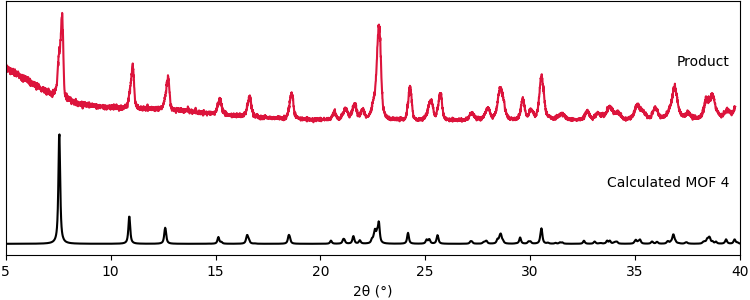


**Figure SI4.** Comparison of the PXRD diffractogram of a batch of Ba-MOF **4** (red, top) with the diagram calculated from single-crystal data (black, bottom).


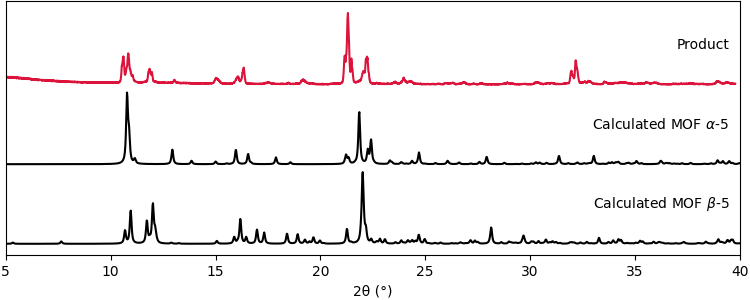


**Figure SI5.** Comparison of the PXRD diffractogram of a batch of Ca-MOF **5** (red, top) with the diagram calculated from single-crystal data for α and β-**5** (black, bottom).


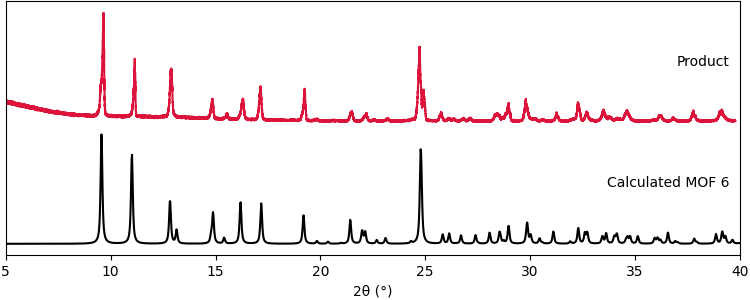


**Figure SI6.** Comparison of the PXRD diffractogram of a batch of Sr-MOF **6** (red, top) with the diagram calculated from single-crystal data (black, bottom).


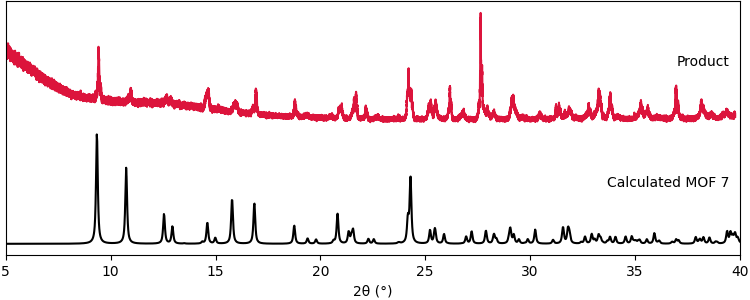


**Figure SI7.** Comparison of the PXRD diffractogram of a batch of Ba-MOF **7** (red, top) with the diagram calculated from single-crystal data (black, bottom).

**Infra-red**


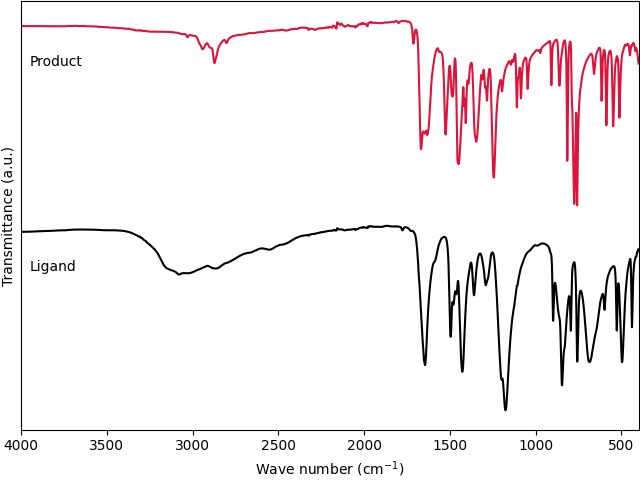


**Figure SI8.** Infra-red spectrum of 2,5-dobdcH_4_ (black) and Mg-MOF **1** (red).


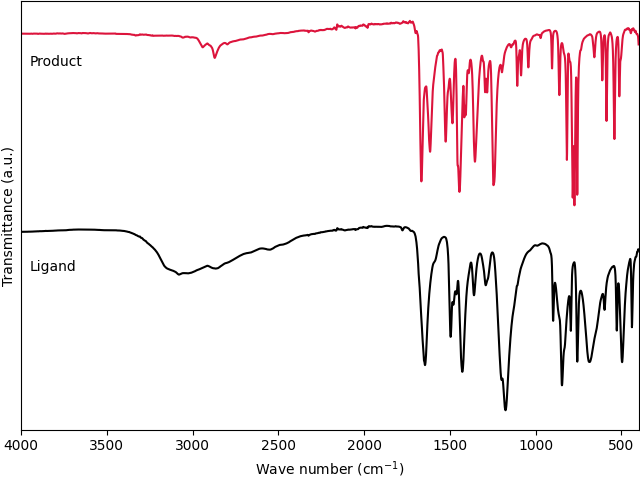


**Figure SI9.** Infra-red spectrum of 2,5-dobdcH_4_ (black) and Ca-MOF **2** (red).


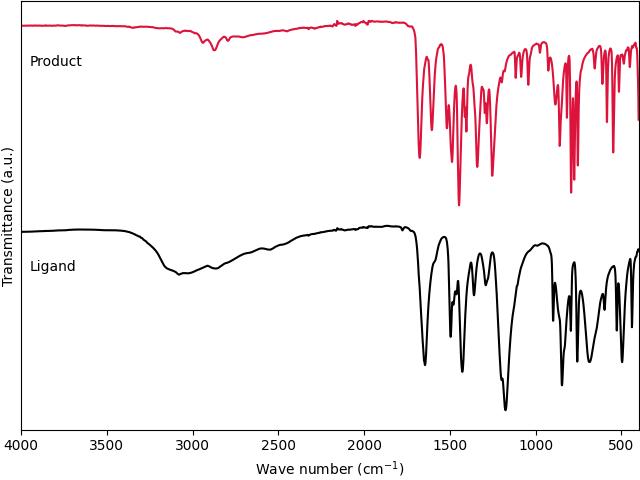


**Figure SI10.** Infra-red spectrum of 2,5-dobdcH_4_ (black) and the batch of Sr-MOF **3** (red).


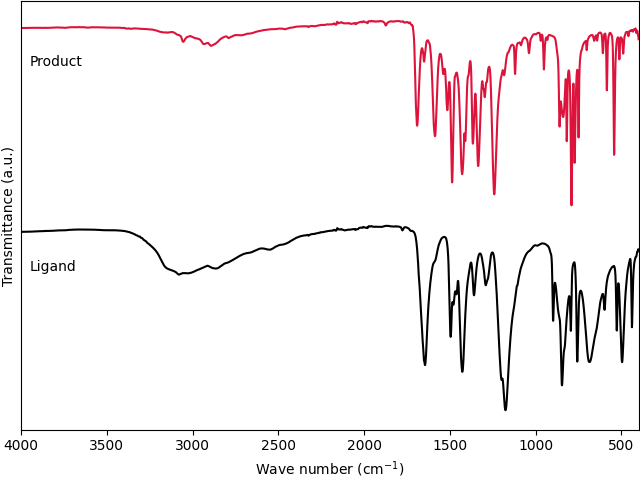


**Figure SI11.** Infra-red spectrum of 2,5-dobdcH_4_ (black) and Ba-MOF **4** (red).


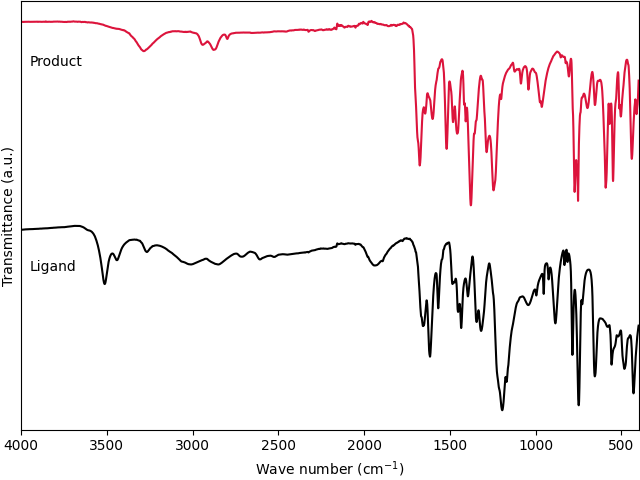


**Figure SI12.** Infra-red spectrum of 2,3-dobdcH_4_ (black) and the batch of Ca-MOF **5** (red).


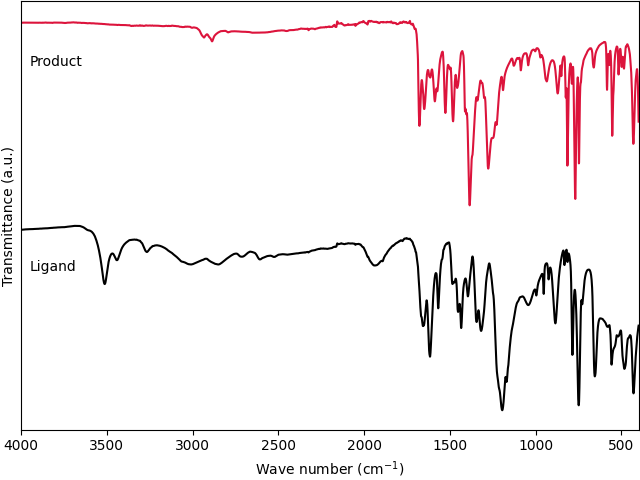


**Figure SI13.** Infra-red spectrum of 2,3-dobdcH_4_ (black) and Sr-MOF **6** (red).


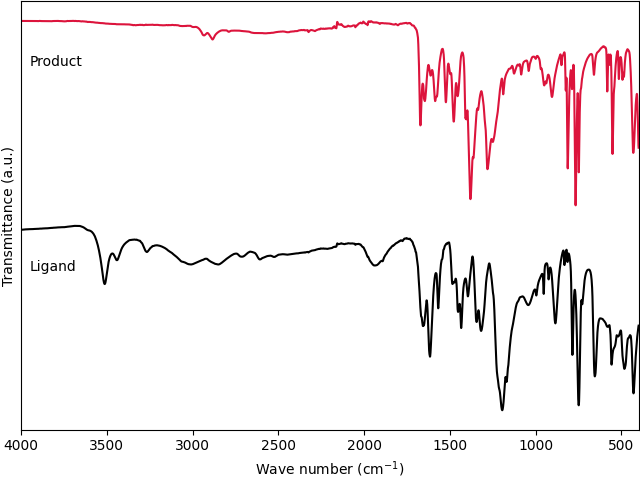


**Figure SI14.** Infra-red spectrum of 2,3-dobdcH_4_ (black) and Ba-MOF **7** (red).

**Thermogravimetric analyses (TGA)**


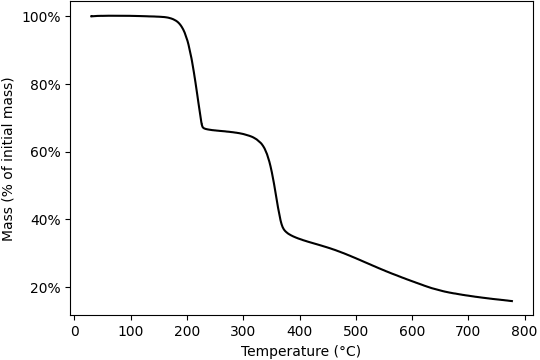


**Figure SI15.** TGA for Mg-MOF **1.**


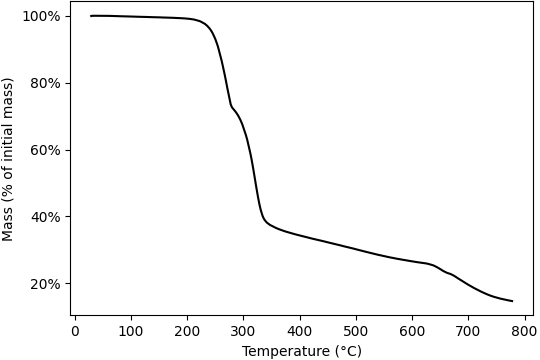


**Figure SI16.** TGA for Ca-MOF **2.**


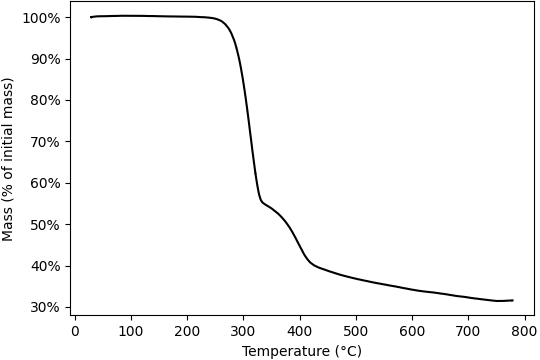


**Figure SI17.** TGA for a batch Sr-MOF **3.**


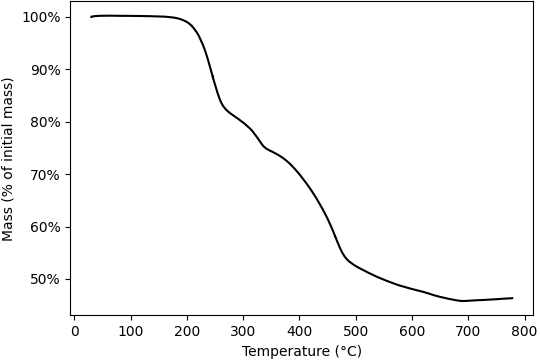


**Figure SI18.** TGA for Ba-MOF **4.**


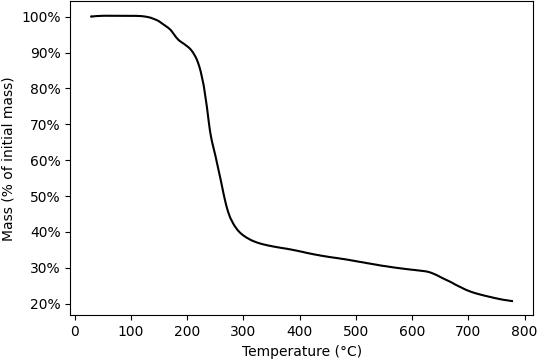


**Figure SI19.** TGA for a batch Ca-MOF **5.**


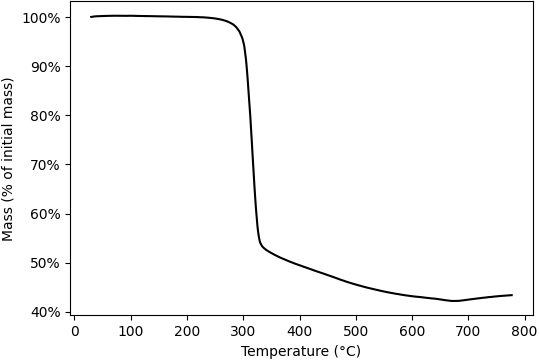


**Figure SI20.** TGA for Sr-MOF **6.**


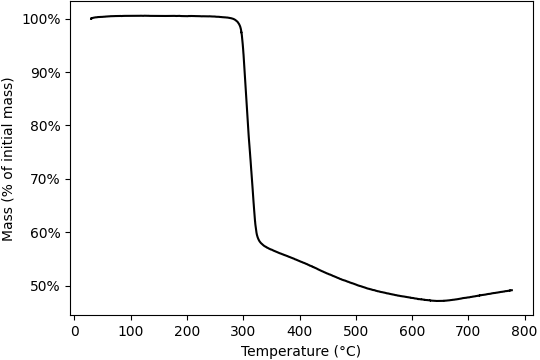


**Figure SI21.** TGA for Ba-MOF **7.**


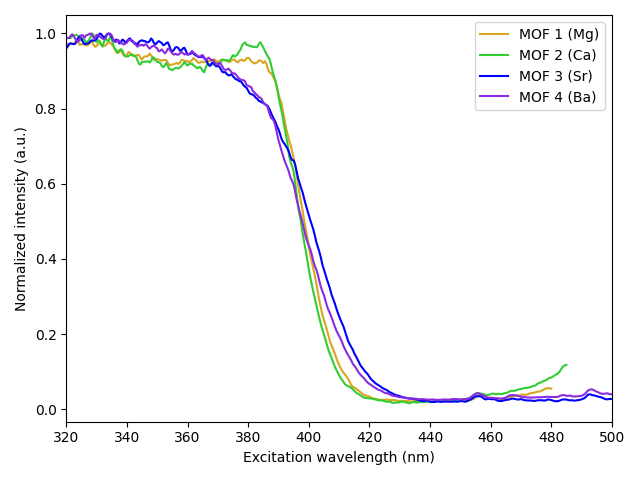


**Figure SI22.** Excitation spectra of MOFs **1**-**4** (λ_em_ = 495 nm for **1**; 500 nm for **2**; 565 nm for **3** and 555 nm for **4**).


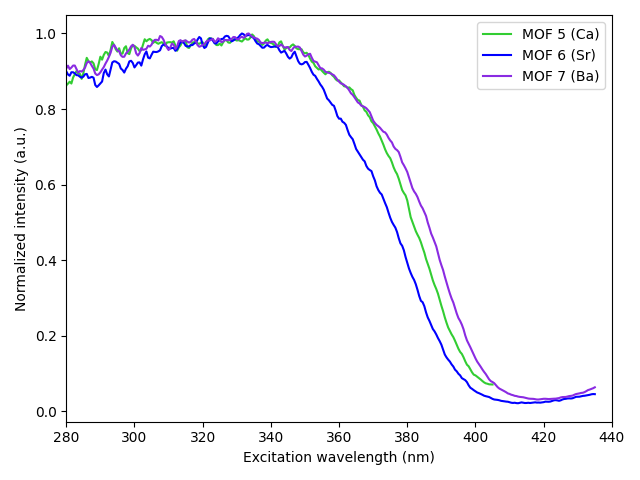


**Figure SI23.** Excitation spectra of MOFs **5**-**7** (λ_em_ = 435 nm for **4**; 450 nm for **6** and **7**).


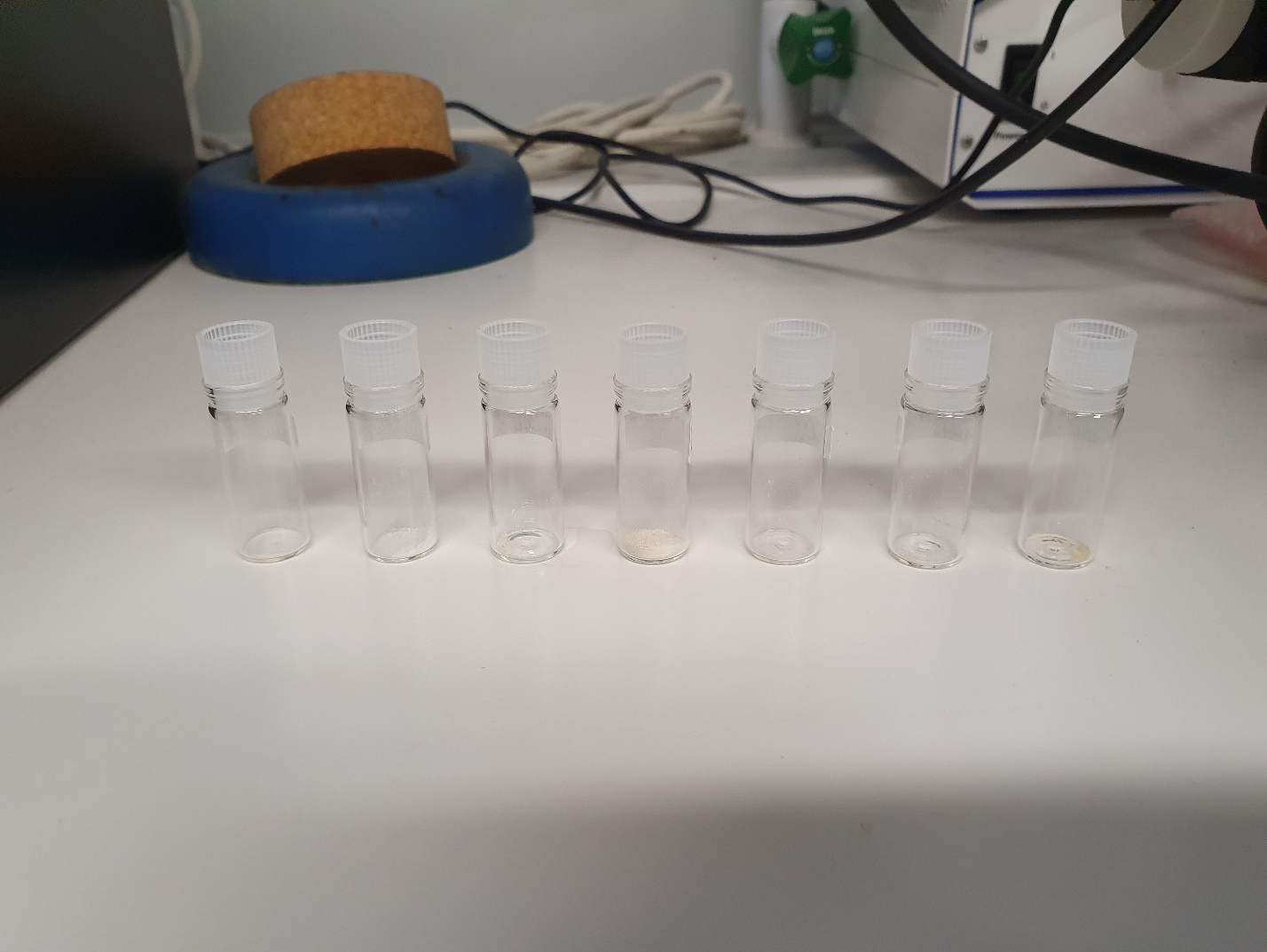


**MOF 7**

**MOF 6**

**MOF 5**

**MOF 4**

**MOF 3**

**MOF 2**

**MOF 1**

**MOF 2**


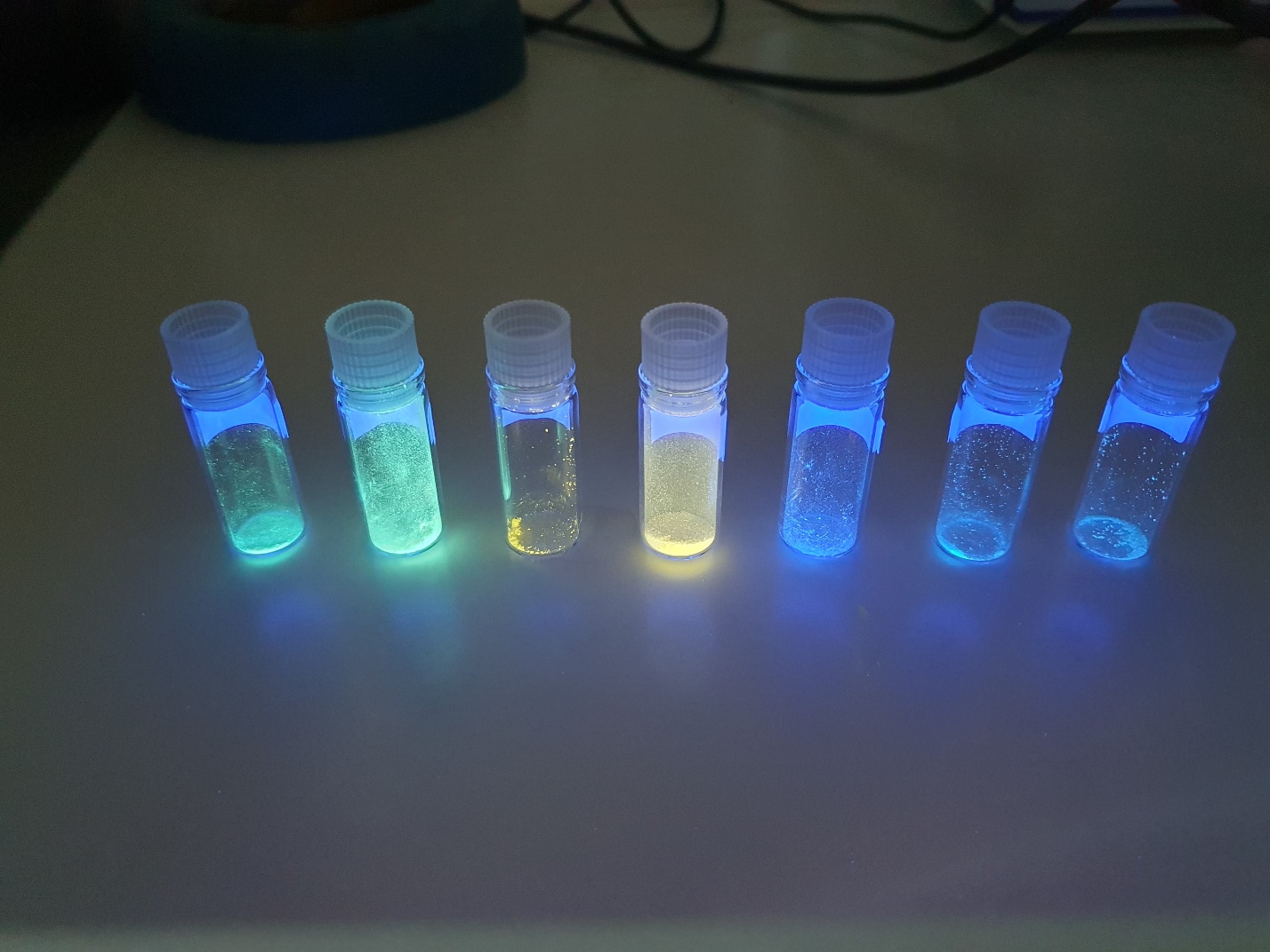


**MOF 5**

**MOF 7**

**MOF 6**

**MOF 4**

**MOF 3**

**MOF 1**

**Figure SI24.** Photographs of MOFs **1**-**7** under ambient light (top) and under a 365 nm UV light (bottom)


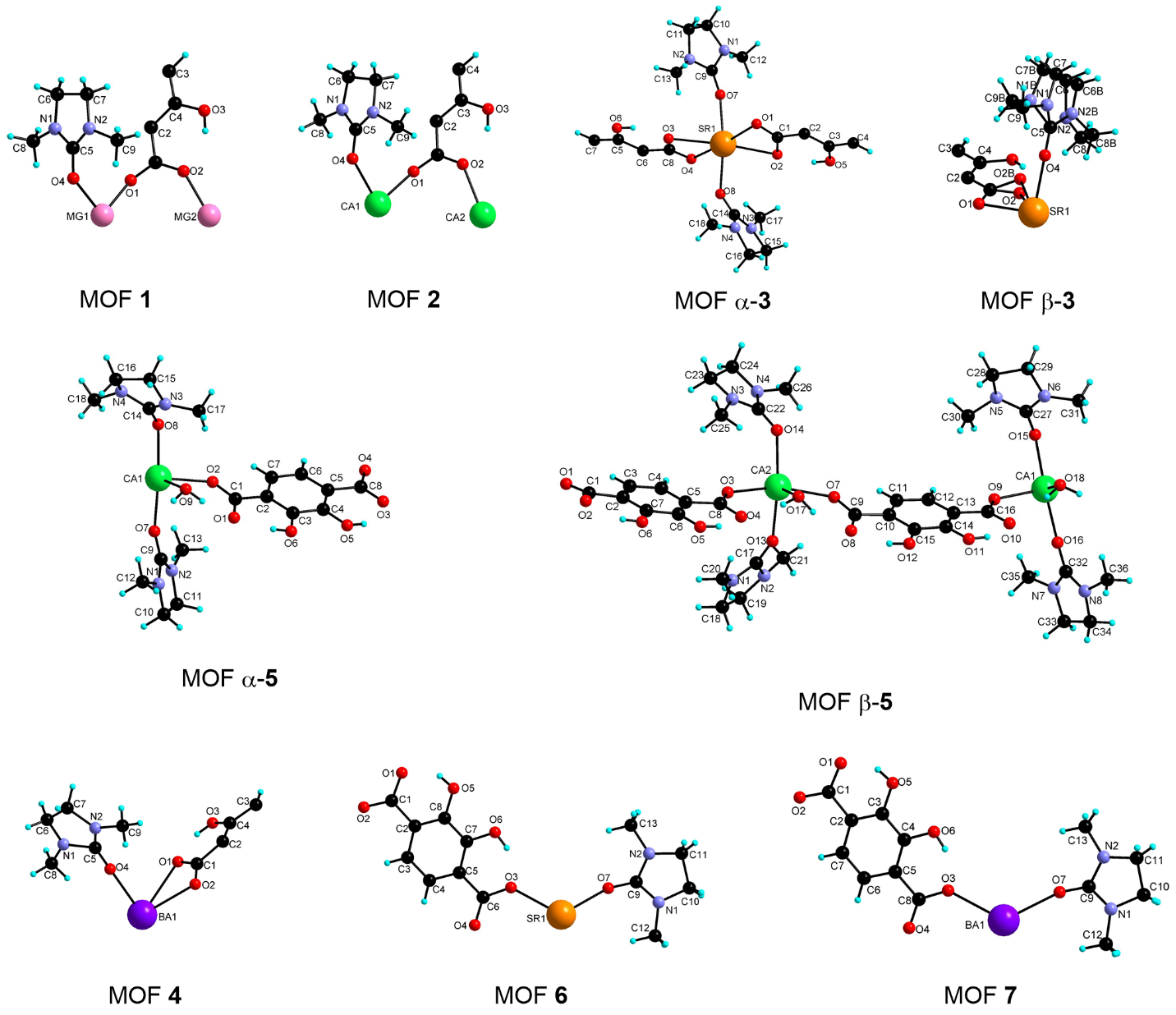


**Figure SI25.** Representation and atom numbering of the asymmetric units of the crystal structures of MOFs **1**-**7**.
